# Supplementary material for: The genome sequence of the hybotid fly, Hybos culiciformis (Fabricius, 1775) (Diptera: Hybotidae)
Source: Wellcome Open Res. 2025 Nov 5;10:617. [Version 1] doi: 10.12688/wellcomeopenres.25056.1 (PMC12744202; doi:10.12688/wellcomeopenres.25056.1)
Supplement: Peer review history

# Reviewer report: Reviewer response for version 1

**DOI:** 10.21956/wellcomeopenres.27622.r138821

**Published:** 2025-12-27

**Authors:** Saverio Brogna

**Affiliations:** 1 University of Birmingham, Birmingham, England, UK

This work presents the genome assembly of a species of Hybos (Diptera). The genome sequence, together with the Hi-C data, is of high quality and provides valuable information. Overall, this represents a valuable contribution to the Darwin Tree of Life project.

 I have only two minor comments for the authors. It would have been useful to perform RNA sequencing from the same specimen (particularly in light of the very low AT content of the genome), and it would have been informative to specify which tissue(s) were used.

Are sufficient details of methods and materials provided to allow replication by others?

Yes

Is the rationale for creating the dataset(s) clearly described?

Yes

Are the datasets clearly presented in a useable and accessible format?

Yes

Are the protocols appropriate and is the work technically sound?

Yes

Reviewer Expertise:

My research focuses on gene expression in eukaryotes, using mostly Drosophila as a model organism. I also have an interest in molecular evolution of genes.

I confirm that I have read this submission and believe that I have an appropriate level of expertise to confirm that it is of an acceptable scientific standard.

# Reviewer report: Reviewer response for version 1

**DOI:** 10.21956/wellcomeopenres.27622.r139739

**Published:** 2025-12-26

**Authors:** Jean-Baka Domelevo Entfellner

**Affiliations:** 1 Data and Research Methods Unit, International Livestock Research Institute (Ringgold ID: 54661), Nairobi, Nairobi County, Kenya

This work presents the first chromosome-level assembly for a dipteran insect, *Hybos culiciformis*. The assembly was performed using adequate sequencing and genome assembly technologies: the authors used PacBio HiFi reads as well as a Hi-C technique for chromosome conformation mapping. The results are correctly presented and overall, there is a good amount of detail on the samples used as well as the methodology followed.

 Nevertheless, I want to point a few flaws that need fixing.

 (1) On the sex of the specimens, there is some amount of uncertainty. The text says that idHybCuli1 (which was used for PacBio sequencing) is “an adult male” (section “Sample acquisition and DNA barcoding”), while the biosample record says that the sex was “NOT COLLECTED” (see e.g. https://www.ebi.ac.uk/ena/browser/view/SAMEA7746460 or https://tolqc.cog.sanger.ac.uk/darwin/insects/Hybos_culiciformis). More importantly, the second specimen, idHybCuli3 (which was used for the Hi-C work) appears to be a **female**. At least, its biosample is registered in ENA as such ( https://www.ebi.ac.uk/biosamples/samples/SAMEA113425977). This discrepancy and the uncertainty about the sex of the specimens used makes the reader feel a bit uncomfortable, especially since the allegedly assembled X and Y chromosomes are extremely short (respectively 3.21 Mb and 0.53 Mb). I am no expert of sex chromosomes in insects, but such values are surprising to me, especially since they are not contrasted with similar or diverging values obtained on closely-related species.

 Of course, it would have been best to run both the PacBio and the Hi-C work on tissue from the same individual, but we understand that operational constraints may have led to the necessity to use two different individuals. Yet, since proximity maps are essential here to confirm the scaffolding results from PacBio reads assembled with YaHS, one would hope that at least, same-sex individuals were used in this study.

 In Figure 3, the authors present the Hi-C contact map, but unfortunately, there is no zoom-in on the scaffolds corresponding to the alleged sex chromosomes. In the absence of any form of comparison to sex chromosomes from other Dipterans, I believe the characterisation of the X and Y scaffolds here is insufficient. The authors only state: “Chromosomes X and Y were assigned based on read and HiC coverage data.” If any form of comparative genomics with other taxa was done, it should be mentioned.

 Also to mention regarding the alleged X and Y chromosomes: while autosomal chromosomes are found to have similar GC-content percentage (“25”, “24.50” and “24” reported in Table 3 -> by the way, I advise the authors to harmonise the presentation of the results in Table 3, with the same precision for all figures), we have markedly different values for X (32%) and Y (28.5%). Is this common in insects, to have markedly different CG content for sex vs. autosomal chromosomes? The authors did remove bacterial contigs using the Assembly Screen for Cobionts and Contaminants (ASCC) after assembly, but some bacteria, fungi or viruses may have failed to be detected by the pipeline. The 0.53 Mb scaffold could very well be made of such foreign bacterial content. Upon zooming in onto the snail plot in Figure 5, in the area corresponding to the scaffolds beyond the first three, the way lines in the blue crown (the one for GC content) are a further indication of potential foreign material.

 (2) On the number of chromosomes found, I would expect a bit of a discussion (even though I understand this is only a “data note” paper). Dipterans most commonly have n=6. Unfortunately, the authors of (Ref no. 2) didn’t study Hybotidae, so one cannot know for sure, but overall, I would welcome at least a small paragraph contrasting this genome data with one or two closest sequenced species.

 (3) Let me now mention some other points, mistakes and things that call for clarification.

 3.1 In the second paragraph of section “Sample acquisition and DNA barcoding”, one can read: “A small sample was dissected from the specimen and stored in ethanol”. We wonder why, and which part of the insect that was.

 3.2 In the first paragraph of section “PacBio HiFi library preparation and sequencing”, one can read: “Prior to library preparation, the DNA was fragmented to ~10 kb.” The reader is puzzled, since it seems it was already the case arising from the earlier use of the Covaris G-tube (“We used centrifuge-mediated fragmentation to produce DNA fragments in the 8-10 kb range”, in the paragraph immediately above). Please clarify whether the two chunks of text refer to the same operation, of if two successive rounds of fragmentation were carried out, in which case, the reader is allowed to ask why such was done.

 3.3 In “The DNA was evenly divided into two aliquots for dual PCR (reactions A and B), both following the manufacturer’s protocol”, it is unclear which manufacturer we are talking about. One guesses “PacBio”, but perhaps it’s better to name them here, since it is in a new paragraph.

 3.4 There seems to be a typo in the text, on “with a heterozygosity of 0.59% and repeat content of 59.85%”. The GenomeScope profile in Figure 2 gives a percentage of unique sequences at 41.1%. Since 100 - 41.1 = 58.9, I guess the authors meant to write in the text “58.85%” and not “59.85%”, pertaining to the repeat content.

 3.5 In Table 2 and in the text (“These chromosome-level scaffolds, confirmed by Hi-C data, are named […]”), whether the output of the assembly based on PacBio reads only yielded the 1,723 contigs in 568 scaffolds. This is my understanding, but the figures in Table 2 could also include some further scaffolding based on the contact maps obtained from the Hi-C data. Please clarify around the word “confirmed” that could also be “obtained”, in the above-mentioned chunk of text.

 3.6 The number of chromosomes set to 5 (and, especially, the issue around the sex chromosomes) is a bit “shaky” if the only consideration leading to this number was “we clearly got three autosomal chromosomes over 10 Mb each, and then we assign X and Y to the following two scaffolds ranked in decreasing order of length”. The sex chromosome system may be more complex (see e.g. Ref no.1)

 3.7 I suggest the authors harmonise the many occurrences of “Hi-C” and “HiC” throughout the text, into a single, constant spelling. I prefer “Hi-C”.

 3.8 I suggest the authors include in Table 3 the data pertaining to the assembled mitochondrion.

 3.9 The curves in Figure 4 seem to signal a higher percentage of heterozygosity than the one reported in Figure 2. The difference is that in Figure 4, the k-mer appearing in reads that were not assembled in the 5 scaffolds (?) are rejected into the black curve on the left, but a quick back-of-the envelope calculation I performed based on the green, red and blue curves from Figure 4 yields much higher a percentage of heterozygosity than the 0.586% reported by GenomeScope 2 in Figure 2. Happy to discuss this further with the authors, as I think this deserves a bit of further examination, and readers would be glad to have a final, ascertained figure for the heterozygosity in this genome assembly.

 3.10 In the first paragraph under “Hi-C library preparation and sequencing”, it is not clear (at least to readers deprived of expertise on the specific protocol used) why you build several libraries, to then pool them in equimolar concentrations. A word of explanation on this would be welcome.

 3.11 Just before the “Genome assembly” section, when authors mention the Illumina NovaSeq 6000, please add a mention of the specific Illumina reagent kit used (e.g. an SP, S1, S2 or S4 flow cell). Related to this, readers can be surprised to see that authors sequenced their Hi-C library to such a high depth (112.01 Gb, i.e. some 266x of the tentative genome size given by GenoScope 2, and some 327x of the final reported genome size). 

 3.12 In the first paragraph under “Assembly quality assessment”, “databases” to be modified to “ **database**” (there is only one database of k-mers associated with a fixed k=31).

 3.13 In the first paragraph under “Genome annotation report”, the authors should qualify the number of transcripts they got from the automated Ensemble annotation. Since no RNA-Seq experiment was conducted here, I suggest writing “21 343 **predicted** mRNAs” rather than “21 343 transcribed mRNAs”.

Are sufficient details of methods and materials provided to allow replication by others?

Partly

Is the rationale for creating the dataset(s) clearly described?

Yes

Are the datasets clearly presented in a useable and accessible format?

Yes

Are the protocols appropriate and is the work technically sound?

Yes

Reviewer Expertise:

bioinformatics, long-read genomics, phylogenetics

I confirm that I have read this submission and believe that I have an appropriate level of expertise to confirm that it is of an acceptable scientific standard, however I have significant reservations, as outlined above.

## References

[1] : Numerous Transitions of Sex Chromosomes in Diptera. *PLOS Biology* .2015;13(4) : 10.1371/journal.pbio.1002078 10.1371/journal.pbio.1002078 PMC440010225879221

[2] : Diptera and Drosophila Karyotype Databases: A Useful Dataset to Guide Evolutionary and Genomic Studies. *Frontiers in Ecology and Evolution* .2022;10: 10.3389/fevo.2022.832378 10.3389/fevo.2022.832378 PMC955580936238425

# Reviewer report: Reviewer response for version 1

**DOI:** 10.21956/wellcomeopenres.27622.r139738

**Published:** 2025-12-11

**Authors:** Steven Fiddaman

**Affiliations:** 1 University of Oxford, Oxford, England, UK

This Data Note presents a chromosome-level genome assembly for the hybotid fly, *Hybos culiciformis*, as part of the Darwin Tree of Life project. Overall, the data provided is comprehensive, and the assembly quality is high.

 The methodology for this approach to genome sequencing is well established and appears to have been followed correctly. 

 I only have a couple of very minor points:

 1. Methods/Sample acquisition and DNA barcoding: “the tissue was lysed” – by what method? Mechanical disruption or chemical dissociation?. 

 2. Methods/Sample acquisition and DNA barcoding:”COI” not defined. 

 3. Methods/Sample acquisition and DNA barcoding:”BOLD” not defined.

Are sufficient details of methods and materials provided to allow replication by others?

Yes

Is the rationale for creating the dataset(s) clearly described?

Yes

Are the datasets clearly presented in a useable and accessible format?

Yes

Are the protocols appropriate and is the work technically sound?

Yes

Reviewer Expertise:

Genomics, genetics, immunogenetics, immunology, virology

I confirm that I have read this submission and believe that I have an appropriate level of expertise to confirm that it is of an acceptable scientific standard.
